# Clinical, molecular and glycophenotype insights in SLC39A8-CDG

**DOI:** 10.1186/s13023-021-01941-y

**Published:** 2021-07-10

**Authors:** Eleonora Bonaventura, Rita Barone, Luisa Sturiale, Rosa Pasquariello, Maria Grazia Alessandrì, Anna Maria Pinto, Alessandra Renieri, Celeste Panteghini, Barbara Garavaglia, Giovanni Cioni, Roberta Battini

**Affiliations:** 1 Department of Developmental Neuroscience, IRCCS Fondazione Stella Maris, Viale del Tirreno 331, Calambrone, 56128 Pisa, Italy; 2 Department of Clinical and Experimental Medicine, University of Pisa, Pisa, Italy; 3 Department of Clinical and Experimental Medicine-Child Neuropsychiatry Section, University of Catania, Catania, Italy; 4 CNR, Institute for Polymers, Composites and Biomaterials, IPCB, Catania, Italy; 5 Department of Medical Genetics, University of Siena, Siena, Italy; 6 Department of Medical Biotechnologies, Med Biotech Hub and Competence Center, University of Siena, Siena, Italy; 7 Department of Molecular Neurogenetics, Fondazione IRCCS Istituto Neurologico “Carlo Besta”, Milan, Italy

**Keywords:** SLC39A8, Congenital disorder of glycosylation (CDG), Movement disorder, Leigh syndrome-like, MALDI-MS, Serum N-glycomics

## Abstract

**Background:**

*SLC39A8*, a gene located on chromosome 4q24, encodes for the manganese (Mn) transporter ZIP8 and its detrimental variants cause a type 2 congenital disorder of glycosylation (CDG). The common *SLC39A8* missense variant *A391T* is associated with increased risk for multiple neurological and systemic disorders and with decreased serum Mn. Patients with SLC39A8-CDG present with different clinical and neuroradiological features linked to variable transferrin glycosylation profile. Galactose and Mn supplementation therapy results in the biochemical and clinical amelioration of treated patients.

**Results:**

Here, we report clinical manifestations, neuroradiological features and glycophenotypes associated with novel *SLC39A8* variants (*c.1048G* > *A*; *p.Gly350Arg* and *c.131C* > *G*; *p.Ser44Trp*) in two siblings of the same Italian family. Furthermore, we describe a third patient with overlapping clinical features harbouring the homozygous missense variant *A391T.* The clinical phenotype of the three patients was characterized by severe developmental disability, dystonic postural pattern and dyskinesia with a more severe progression of the disease in the two affected siblings. Neuroimaging showed a Leigh syndrome-like pattern involving the basal ganglia, thalami and white matter. In the two siblings, atrophic cerebral and cerebellum changes consistent with SLC39A8-CDG were detected as well. Serum transferrin isoelectric focusing (IEF) yielded variable results with slight increase of trisialotransferrin isoforms or even normal pattern. MALDI-MS showed the presence of hypogalactosylated transferrin N-glycans, spontaneously decreasing during the disease course, only in one affected sibling. Total serum N-glycome depicted a distinct pattern for the three patients, with increased levels of undergalactosylated and undersialylated precursors of fully sialylated biantennary glycans, including the monosialo-monogalacto-biantennary species A2G1S1.

**Conclusions:**

Clinical, MRI and glycosylation features of patients are consistent with SLC39A8-CDG. We document two novel variants associated with Leigh syndrome-like disease presentation of SLC39A8-CDG. We show, for the first time, a severe neurological phenotype overlapping with that described for SLC39A8-CDG in association with the homozygous *A391T* missense variant. We observed a spontaneous amelioration of transferrin N-glycome, highlighting the efficacy of MS-based serum glycomics as auxiliary tool for the diagnosis and clinical management of therapy response in patients with SLC39A8-CDG. Further studies are needed to analyse more in depth the influence of *SLC39A8* variants, including the common missense variant, on the expression and function of ZIP8 protein, and their impact on clinical, biochemical and neuroradiological features.

**Supplementary Information:**

The online version contains supplementary material available at 10.1186/s13023-021-01941-y.

## Introduction

*SLC39A8*, a gene located on chromosome 4q24, encodes for the manganese (Mn) transporter ZIP8, which plays a major role in regulating Mn homeostasis in blood and tissues [1]. Mn is an essential trace element and has a major role as cofactor for various enzymes (e.g. pyruvate carboxylase, arginase, superoxide dismutase-2, glutamine synthase and glycosyltransferases) [2, 3]_._

Since Mn is a cofactor for beta-1,4-galactosyltransferase [2], *SLC39A8* variants are associated with a congenital disorder of glycosylation (CDG), characterized by deficient galactosylation of glycan chains of glycoproteins. Moreover, the common *SLC39A8* variant rs13107325 (C → T) in exon 8 (*A391T)*, is associated with increased risk for other neurological and systemic disorders, and with decreased serum Mn.

CDG are inborn errors of metabolism characterized by improper glycosylation of glycoconjugates. Protein glycosylation defects are caused by genetic variants in the N- and/or O-glycosylation pathways. Serum transferrin isoelectric focusing (IEF) and capillary electrophoresis (CDT) are the conventional laboratory tests for the N-glycosylation defects associated with sialic acid deficiency. These blood measurements enable to distinguish between CDG-I (cytosol and endoplasmic reticulum defects of N-glycan assembly) and CDG-II (Golgi processing defects). So far fourteen subjects with SLC39A8-CDG have been reported [2–5]. Most patients exhibited a serum Tf IEF type 2 pattern and/or low to undetectable blood Mn levels and elevated Mn urine levels, consistent with renal wasting [3, 6]. The variability in Mn blood levels among the affected patients is likely due to partial compensation by other transporters, to different effects of SLC39A8 variants on Mn homeostasis and to variations of Mn levels as result of the dietary intake [3].

SLC39A8-CDG presents with developmental delay, severe intellectual disability, seizures, and cerebral and/or cerebellar atrophy [6]. Dystonia and basal ganglia T2-hyperintensities on magnetic resonance imaging (MRI) are also part of the neurological spectrum [4, 5]. SLC39A8-CDG features have been related to both Mn deficiency and hypoglycosylation [2]. In particular, SLC39A8-CDG may present with the clinical, biochemical and neuroradiological features of Leigh syndrome-like mitochondrial disease, probably due to reduced activity of Mn-dependent superoxide dismutase (SOD), a reactive species scavenger in mitochondria [4]. Since hypoglycosylation in SLC39A8 deficiency is secondary to a defect of galactosylation, galactose supplementation (in combination with uridine) was reported to improve transferrin glycosylation [2, 4]. More recently, Mn-II-sulfate supplementation was found to ameliorate both biochemical (e.g., improvement of glycosylation of serum transferrin and normalization of blood and urinary Mn levels) and clinical features (e.g. improved motor abilities, ataxia, muscle strength, postural control, frequency and severity of epilepsy, vision, hearing and swallowing) [7]. Appropriate Mn supplementation was not associated with clinical or neuroradiological signs of manganism (e.g. psychiatric symptoms or Mn deposition in basal ganglia with associated dystonia) [7]; however, close monitoring of the patients is recommended. Transferrin glycosylation analyses may represent a useful marker to establish the therapeutic dose of Mn [7].

Due to the paucity of studies on clinical, glycosylation and molecular correlates of SLC39A8-CDG, further insights are warranted to better characterize this disorder, also in light of possible therapeutic interventions.

## Aims

We describe clinical and neuroradiological features, and the glycophenotypes associated with novel *SLC39A8* variants (Family 1, Patient-1 and Patient-2) and with the already known *SLC39A8* homozygous missense variant *p.Ala391Thr* (Family 2, Patient-3)*.*

## Methods

The study was based solely on information and investigations that were carried out as part of the routine clinical care of CDG patients. Informed consent was signed by the parents.

### Molecular investigations

Next Generation sequence (NGS) analyses were undertaken in study patients to elucidate underlying mechanisms of severe generalized dystonia and global developmental delay.

Array-CGH analysis was performed on an Agilent 60 K platform with a resolution of 100 kb. Mitochondrial gene analyses (e.g. *MTDN1, MTDN2, MTDN3, MTDN4L, MTDN4, MTDN5, MTDN6, ECHS1*) (Patient-1) were performed by use of PCR-amplification and direct sequencing of PCR-amplified DNA. Whole mitochondrial genome sequencing (Patient-3) was performed by massive parallel sequencing using NexteraXT technology (Illumina, San Diego, CA). CDG panel analysis (Patient-1) was performed by massive parallel sequencing covering all exons and their flanking intronic regions (± 20 bp) for 79 genes associated with CDG-I or CDG-II and for a few genes related to congenital muscular dystrophies. *PLA2G6* and *ECHS1* gene sequence analysis was obtained by using PCR amplification and Direct Sequencing through the automated DNA sequencer 3100 Genetic Analyzer ABI Prism). Comprehensive analysis of the NGS panel for 35 genes associated with dystonia (Patient-1) was performed by using NGS Sequencer Illumina MiSeq. Genome wide analysis (Patient-2) was performed through Proton Ion Torrent chips. DNA library preparation was performed according to the Ion AmpliSeq Exome Kit. Data analysis was conducted with variant Caller version 5.2.0.34 plugin from Ion Torrent Suite software (v.5.2.0), which requires a minimal minor allele frequency of 0.1 and a minimal coverage of 20 ×.

SLC39A8 variants were confirmed by Sanger sequencing in all three patients.

### Glycosylation analyses

N-glycosylation analyses were undertaken in all study patients in the context of the metabolic diagnostic procedure for dystonia and subsequently to endorse the glycophenotype associated with studied SLC39A8-CDG variants.

#### Serum glycosylation analyses

Glycosylation analyses were performed by quantitation of intact transferrin isoforms through isoelectric focusing (IEF) and/or capillary zone electrophoresis (CZE) using the carbohydrate-deficient transferrin (CDT) test. Transferrin isoforms percentage was compared with internal reference values. In-depth transferrin and total serum N-glycan structural analyses were conducted by matrix-assisted laser desorption/ionization time-of-flight (MALDI-TOF) mass spectrometry (MS). In this case, transferrin was isolated from serum by immunoaffinity depletion on IgY microbead spin columns (Seppro™ GenWay Biotech, San Diego, CA) and deglycosylated using PNGase F [8]. Transferrin N-glycans from the patient and age-matched controls’ sera were analyzed after sample permethylation [9]. Total N-glycan profiling was obtained by using 10 μL of serum as previously described [8]. Briefly, proteins were denatured with *Rapi*Gest™ SF surfactant, reduced and alkylated with DTT and IAA respectively, then deglycosylated by PNGase F. Released N-glycans were permethylated prior to MALDI-TOF MS analysis, performed in reflector mode and in positive polarity on a 4800 Proteomic Analyzer (AB Sciex). Total N-glycans spectra from age-matched control sera were used for comparison.

## Results

### Patients’ phenotype

#### Family-1

Patient-1 and -2 were sibs, born to healthy unrelated Italian parents.

Patient-1 is a male born at term after an uncomplicated pregnancy. Length and weight at birth were within the normal range. Parental concerns were initially raised at 4 months of age when, after a post-vaccine febrile event, the patient presented with recurrent episodes of post-meal vomiting with poor weight and length gain. Medical evaluation at our department was carried out at 5 months of age. Global delay, gastroesophageal reflux (GER), generalized hypotonia with dystonia movements and stiffening of lower limbs were noted. Brain Magnetic Resonance Imaging (MRI) was normal. Electroencephalogram (EEG) showed slow activity in the posterior areas.

Due to severe and persistent GER with feeding difficulties and poor weight gain, gastrostomy and fundoplication were performed at the age of 11 months.

Since serum CKs were constantly very high (> 1000 U/L), a muscular biopsy was performed and a reduction of respiratory chain complex I activity was found. Electromyography (EMG) was normal, while motor and sensitive nerve conduction velocities (NCV) were characterized by reduced amplitude in all sensory nerves studied. A slight increase of acetoacetic, fumaric and ketoglutaric acids was also identified in urine. A possible mitochondrial disease was investigated and excluded through the analysis of the mitochondrial and nuclear genomes. Furthermore, no pathological changes were found on skin biopsy. MRI was repeated when the patient was 15 months of age, showing a multifocal encephalopathy in the basal ganglia, the thalami, and the periventricular and subcortical white matter. Proton spectroscopy showed an increased lactate peak.

Over the years, the patient remained globally delayed and he developed scoliosis. He suffered multiple infectious episodes of the upper airways that needed to be treated with antibiotics.

**Fig. 1:**
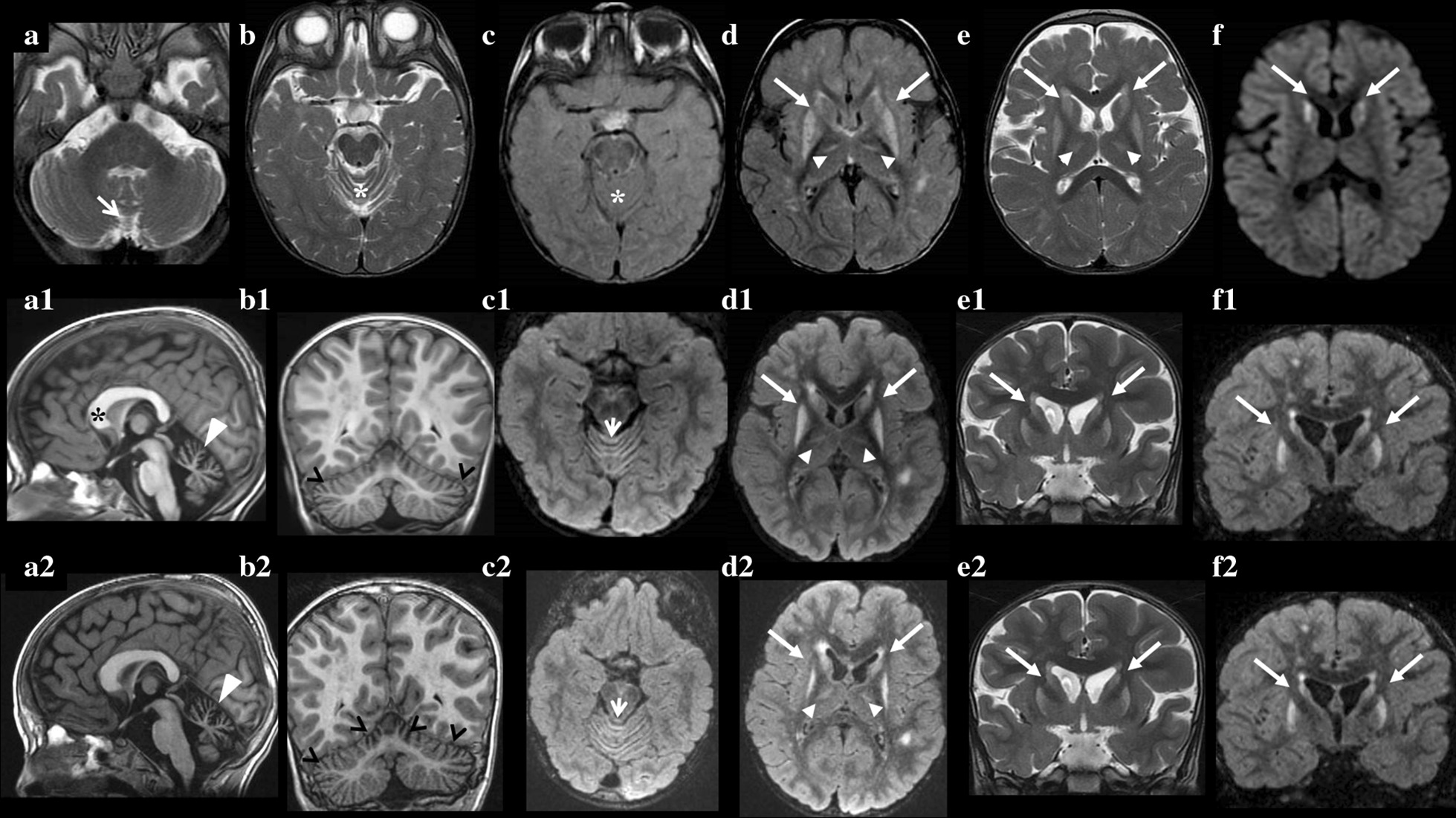
Magnetic Resonance Imaging scans of Patient-1: neuroradiological changes over time. Axial T2 (**a, b**) and FLAIR-weighted (**c**) images show slight enlargement of the superior and posterior vermis space (arrow) without signal alteration (asterisks). Axial FLAIR (**d**) and FSE T2-weighted (**e**) images show bilateral signal alterations of the striatal nuclei (arrows) and thalami (head-arrows) with reduced diffusivity of the head of the caudate nuclei (arrows) in the diffusion weighted image (**f**). The second line of MRI images (**a1, b1, c1, d1, e1, f1**) was performed two years after the first one (**a–f**). Sagittal (**a1**) and coronal (**b1**) T1-weighted images show atrophy of cerebellar vermis, signal alteration of the superior vermis and the initial involvement of cerebellar hemispheres (head-arrows). Sagittal T1-weighted image (**a1**) also shows a dysmorphic corpus callosum, especially for the hypertrophy of the genu (asterisk). Axial FLAIR-weighted (**c1**) images show mild cortical hyperintensity of the superior cerebellar vermis. Axial (**d1**), coronal (**f1**) FLAIR-weighted and coronal T2-weighted (**e1**) images show the same signal alterations and lesions in the supratentorial region of the first line ones (**a–f**), except for a slight reduction in the signal alteration of striatal nuclei, which also appear more swollen (arrows) (**d1**). A lack of restriction of proton diffusivity is also evident. The third line of MRI images (**a2, b2, c2, d2, e2, f2**) was performed 2 years after the second one (**a1, b1, c1, d1, e1, f1**). Sagittal (**a2**) and coronal (**b2**) T1-weighted images show the progression of cerebellar atrophy (head-arrows). An increased signal alteration of the superior vermis is evident in axial FLAIR-weighted image (arrow) (**c2**). (**d2, e2, f2**) images, compared to the same sequence images in the second line (**d1, e1, f1**) show no new signal alteration or lesion of the supratentorial region

Due to a clinical worsening characterized by increased dystonic postures, dyskinetic movements in the oral region, eye rolling, back-arching and involuntary stiffening of lower limbs, brain MRI was repeated again at 4 years of age. In addition to the already known neuroradiological pattern, a new lesion in the superior portion of the cerebellar vermis was detected. The same MRI feature was previously observed in Patient-1’s older sister (Patient-2). Serial MRIs documented a progressive cerebellar atrophy (Fig. 1). Since the age of 5, the child had focal and generalized clonic seizures and atypical absence epilepsy, non-responsive to conventional anti-epileptic drugs. *PLA2G6* and *ECHS1* gene sequence analysis was performed to rule out infantile neuroaxonal dystrophy (INAD) and bilateral striatal necrosis, respectively. Biotinidase activity in dried blood spot was normal. Serum transferrin IEF glycosylation pattern showed a type 2 pattern: an increase of trisialotransferrin, which indicated the lack of one sialic acid unit from the normal tetrasialo-isoform. This finding was explained by the increase of truncated monosialo-biantennary glycans as revealed by MALDI MS analyses. However, no mutations were found in the CDG panel covering 79 genes associated with CDG-I or CDG-II and few genes related to congenital muscular dystrophies.

The last neurological examination showed postural hypotonia, dystonic-dyskinetic movements, drug-resistant epilepsy, and severe intellectual disability. He passed away at age of 10, during an intercurrent infection.

Patient-2 is the older sister of Patient-1. She suffered from a neurodegenerative disease characterized by hypopostural tetraplegia, movement disorder with dystonia and choreoathetosis, and mental disability. Onset of symptoms was at 5 months of age. Brain MRI documented progressive cerebellar atrophy and signal alterations in basal ganglia. Muscle biopsy (electron microscopic and immunohistochemical analysis) and analysis of mitochondrial genome yielded normal results. Transferrin glycosylation analyses showed a mild sialylation defect consisting of a slight increase of the trisialo-component. This alteration of sialylation processes was confirmed by a moderate enhancement of the monosialo-biantennary glycoform and its core fucosylated counterpart by MALDI MS analysis in negative polarity of underivatized transferrin-released glycans (data not shown). Unfortunately, she passed away at the age of 9 years before a definitive diagnosis was reached.

#### Family-2

Patient-3 is the first-born to unrelated Romanian parents. He was born at term after an uneventful pregnancy. Immediately after the delivery, the patient was admitted to the Intensive Care Unit for dyspnoea, desaturation, and myoclonic fits without electroencephalographic correlation. He was discharged in good health.

**Fig. 2:**
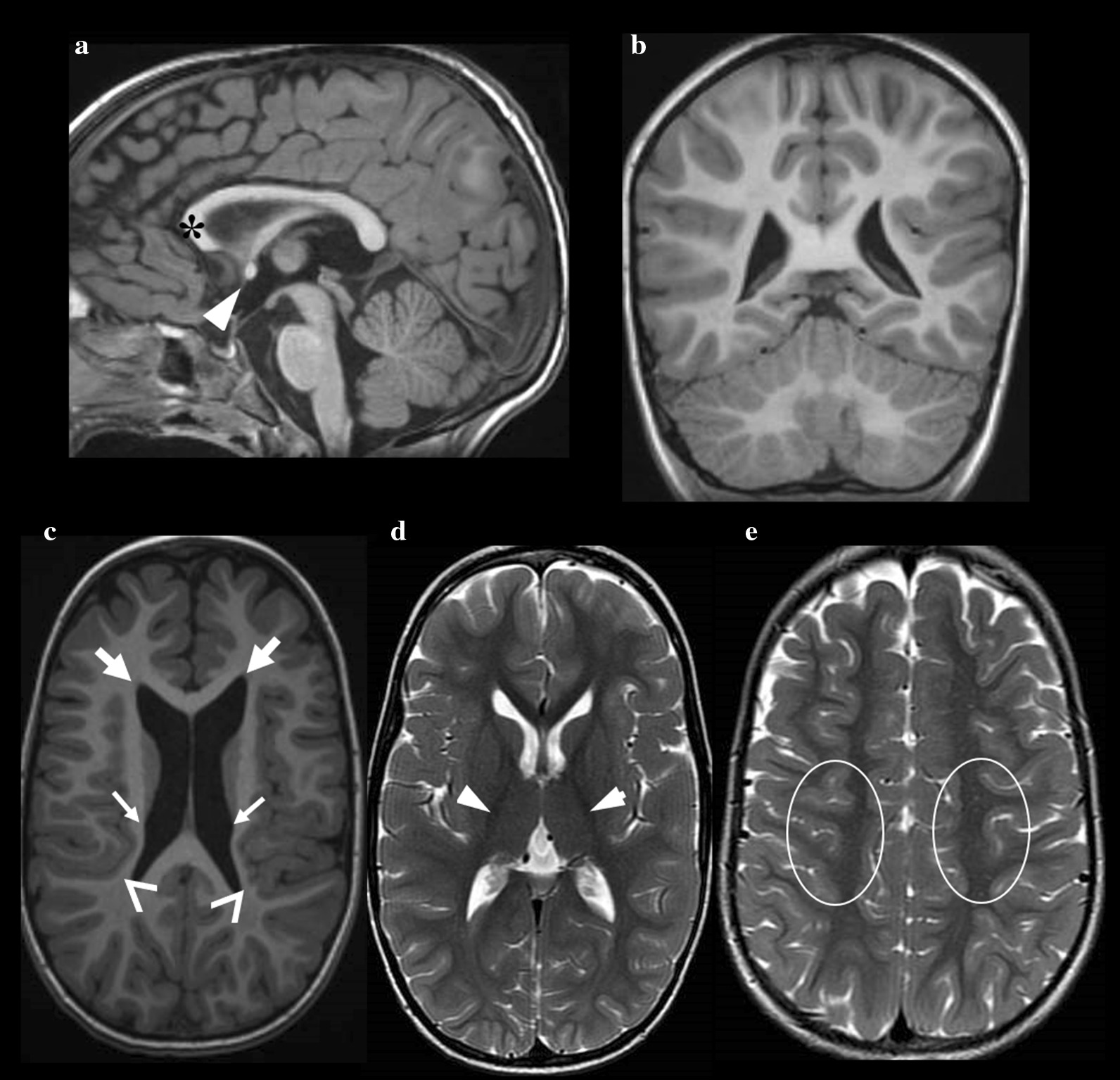
Magnetic Resonance Imaging scans of Patient-3. Sagittal T1-weighted image (**a**) shows a thinning of the genu of the corpus callosum (asterisk) and a slight hypetrophy of the anterior commissure (head-arrow). Scaphocephalic skull is also evident. Sagittal (**a**) and coronal (**b**) T1-weighted images show normal cerebellum. Axial T1-weighted image (**c**) shows dysmorphic features of lateral ventricles with dilatation of the frontal horns (arrow) and irregular ventricular walls (small arrows). A reduction of volume of insulo-temporal white matter (head-arrow) is also evident. Axial T2-weighted images (**d–e**) show normal signal of the white matter at the level of the posterior limb of the internal capsule (head-arrow) and of the paracentral region (oval)

Parental concerns were raised in the third month of age when he displayed a scaphocephalic skull, floppiness, global delay and stunted growth. Brain MRI, performed at various ages, showed white matter thinning in the temporo-parieto-occipital region, thinning of the genu of the corpus callosum and bilateral reduction of thalamic volume (Fig. 2). Spinal MRI was normal. No genomic rearrangement was identified by array-CGH analysis. In the following years, a more comprehensive neurometabolic screening was carried out. A reduction of methionine was found in plasma, urine, and liquor samples. Urinary oligosaccharides and organic acids, plasmatic homocysteine, serum very-long fatty acids and ceruloplasmin were all within the normal range as were neurotransmitter levels in the liquor. Beta-galactocerebrosidase activity on dried blood spot and alpha-fucosidase, alpha-mannosidase and alpha-hexosaminidase assay in leukocytes were all normal. Serum transferrin IEF was normal. Plasma lactic acid was slightly increased. Brainstem evoked potentials and visual evoked potential suggested impaired auditory and visual conduction. Nerve conduction velocity was normal. EEG showed slow activity and sharp waves in the frontal and centro-temporal areas, bilaterally. A muscular biopsy was performed for a suspected mitochondrial disease. Respiratory chain enzymes in skeletal muscle showed a decreased activity of complex I + III and an elevated activity of citrate synthase. However, analyses of mitochondrial and nuclear genes associated with mitochondrial diseases ruled out a primary mitochondrial disorder. Whole exome sequencing (WES) did not detect any significant pathogenic variant. Due to poor weight gain and severe feeding difficulties, gastrostomy and fundoplication were performed when the patient was 4.

Medical evaluation was carried out by our Department when the patient was 5 years-old. Postural hypotonia with poor head control, dystonia, dyskinetic movements in the oral district, eye rolling, pyramidal signs (positive Babinski sign, clonus and hyperelicitable osteotendinous reflexes), divergent strabismus of the right eye and cranial asymmetry were observed. EEG and brain MRI confirmed previous findings. Since the history and the clinical presentation of this patient were very similar, even if less severe, to that of Patient-1, sialotransferrin glycosylation analysis was repeated by capillary zone electrophoresis (CZE) when the patient was 5. A slight increase of the trisialotransferrin isoform was detected.

### *SLC39A8* molecular variants

In Patient-1, the analysis of a gene panel associated with dystonia revealed two novel compound heterozygous variants in the *SLC39A8* gene (ZIP8, OMIM #608732), *c.1048G* > *A* (*p. Gly350Arg*) and c*.131C* > *G* (*p.Ser44Trp*), inherited from the mother and the father, respectively. A similar clinical and neuroradiological picture associated with the same serum transferrin glycan pattern suggested the same diagnosis also for the sister (Patient-2). In this patient, genetic investigations, performed on the DNA obtained from fibroblasts, acquired before death, confirmed the diagnosis. Family-l *SLC39A8* missense variants have not been reported and are not yet annotated on the ClinVar database (https://www.ncbi.nlm.nih.gov/clinvar/); the *c.131C* > *G* has been reported in dbSNP with rs772391167. Their extremely low allele frequency, along with the compound heterozygosity, suggest a likely detrimental effect on protein function. The *p.Gly350Arg* variant is characterized by a Combined Annotation Dependent Depletion (CADD) 34 (https://cadd.gs.washington.edu/snv). The silico prediction sites (Mutation taster, PolyPhen2.0, Provean) investigated consider *p.Gly350Arg* pathological; only the SIFT website supports it as tolerated but with scores at the limit. This variant is absent in gnomAD (genome aggregation database). This variant is located at the position-1 on the 5′ splice site in exon 7. Therefore, it most likely modifies the splicing of this region inducing exon skipping or, with less probability, intron retention. The *p.Ser44Trp* variant is located in exon 2 and has a CADD score of 28.6; investigated silico prediction sites consider it pathological. The variant *p.Ser44Trp* is absent in gnomAD.

As to Family-2, the consistency between the clinical suspicion and the biochemical data, led to a re-evaluation of the exome sequencing, focusing on CDG associated genes. The *SLC39A8* common missense variant *c.1171G* > *A* (*p.Ala391Thr*) was found in homozygosity. The unaffected parents were both carriers of the same variant. Although in Family-2 the variant *p.Ala391Thr* (in exon 8) (rs13107325) has a total allele frequency of 0,04 according to GnomAD database, with a CADD of 34, and is predicted to be likely damaging by 3 out of 6 prediction tools, it affects a highly conserved amino acid residue and it has been previously associated with an increased risk of schizophrenia and other neurological or systemic disorders, modified MRI signal in certain brain regions such as basal ganglia and decreased blood manganese levels in both homozygous and heterozygous carriers (18% and 10% respectively) [16].

### Serum transferrin glycosylation analyses

Transferrin is an abundant serum glycoprotein with two N-glycosylation sites occupied by biantennary disialylated N-glycans. In CDG-I, monoglycosylated (disialo-) and aglycosylated (asialo-) transferrin isoforms increase due to the lack of the N-glycan moiety in one or both protein glycosylation sites. In CDG-II, the typical transferrin glycosylation defect is due to the presence at the glyco-sites of aberrant N-glycan structures lacking terminal sialic acids with increase of undersialylated (tri-, di-, mono- and asialo-) isoforms. In Patient -1 and -3, repeated serum transferrin analyses by IEF and/or CE showed an increase of trisialo- (8–15%; n.v. ≤ 5,5) and decrease of tetrasialo-transferrin (75–82%, n.v. ≥ 84%) indicating a CDG type 2 pattern (Additional file 1: Fig. S1).

### Serum N-glycome analyses by MALDI-TOF MS

Detailed glycosylation analyses both on total serum and on serum transferrin were performed in Patient-1 by MALDI MS analyses at age 3 (during the diagnostic work-up) and at the age of 7 (after the detection of *SLC39A8* variants). The whole serum N-glycosylation analysis was also performed on a blood sample from his affected sister (Patient-2) at the age of 4. The same comprehensive N-glycan analyses of Patient-1 were performed on Patient-3 when he was 5.

**Fig. 3:**
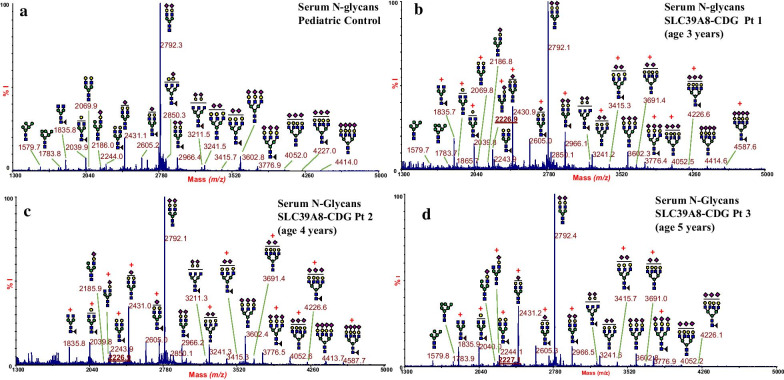
MALDI-TOF MS profiles of permethylated serum N-glycans from the studied SLC39A8-CDG patients compared to a reference control. Serum N-glycan analysis in a paediatric representative control (**a**). N-glycan analyses on this study’s patients, showing increased amount of hyposialylated and/or hypogalactosylated biantennary and triantennary structures and an overall increase in fucosylation (**b–d**). The observed increases are outlined by red marks. Glycan structures were assigned following consortium for functional glycomics guidelines: N-acetylglucosamine, blue square; mannose, green circle; galactose, yellow circle; sialic acid, purple lozenge; fucose, red triangle

In SLC39A8-CDG, galactosylation deficiency is caused by impaired Mn-dependent β1,4-galactosyltransferase (B4GALT) enzyme activity [2]. In human serum glycoproteins reduced B4GALT enzymes activity mainly results in decreased galactosylation of the biantennary asialo, agalacto N-glycans (A2, FA2) to form the corresponding, asialo monogalactosylated structures (A2G1, FA2G1), which, in turn, are further extended to form biantennary monosialo, monogalacto N-glycans (A2G1S1, FA2G1S1). Then, B4GALT adds a second galactose to form the biantennary, digalactosylated monosialo structures (A2G2S1, FA2G2S1) that in turn are elongated to the mature, disialo biantennary N-glycans (A2G2S2, FA2G2S2). Compared to controls (see Fig. 3a as representative control spectrum), MALDI MS analyses of the whole serum N-glycans conducted on the three patients showed common features of defective glycosylation, consisting of an increased amount of hyposialylated biantennary and triantennary structures, and an overall increase in fucosylation (Fig. 3b-d). Notably, the presence of the precursor glycan A2G1S1 (at ~ *m/z* 2227, underlined mass peaks Fig. 3b–d) not detected in the reference controls, was observed in the three patients (Fig. 3a). This glycoform, corresponding to the biantennary truncated N-glycan NeuAc_1_Gal_1_Man_3_GlcNAc_4_ lacking one terminal NeuAc-Gal epitope, has been explicitly addressed to a distinctive serum N-glycome signature for B4GALT deficiency in SLC39A8-CDG patients [5].

**Fig. 4:**
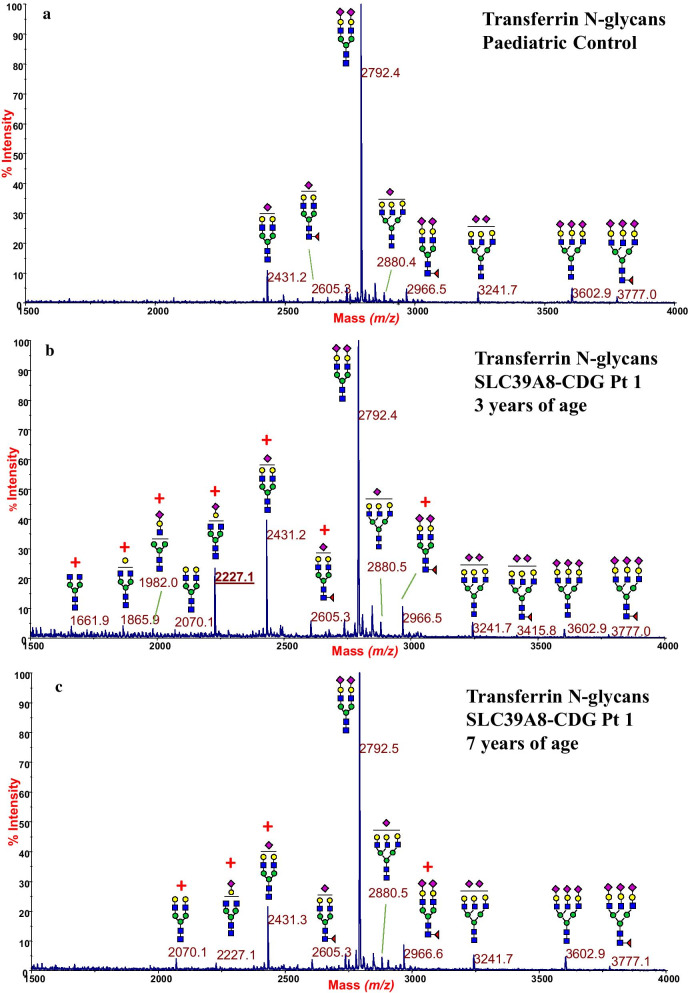
MALDI-TOF mass spectra of permethylated transferrin N-glycans in SLC39A8-CDG Patient-1 compared to a reference control, showing the spontaneous amelioration of the glycosylation profile over a four-years period. Transferrin N-glycan analysis in a paediatric representative control (**a**). Transferrin N-glycan analysis conducted on Patient-1 at 3 years of age (**b**), reveals a marked increase of the monosialo-biantennary glycoform at *m/z* 2431.2 (A2G2S1) and the occurrence of the monosialo- monogalacto-biantennary glycan at *m/z* 2227.1 (A2G1S1). Minor amounts of other truncated glycoforms are also detected. Serum transferrin MALDI-TOF MS analysis of Patient-1 at age of 7 years (**c**) shows a significant spontaneous improvement, with a drastic reduction of all the defective species. Compared to control, the observed glycan increases are outlined by red marks. Glycan structures were assigned following consortium for functional glycomics guidelines: N-acetylglucosamine, blue square; mannose, green circle; galactose, yellow circle; sialic acid, purple lozenge; fucose, red triangle

Conversely, serum transferrin N-glycan analyses, performed in Patient-1 and -3, did not show a typical pattern. The transferrin glycosylation analysis performed in Patient-3 showed only very slight unspecific glycosylation changes, such as minor increases in hyposialylation and fucosylation (Additional file 2: Fig. S2), whereas the transferrin N-glycan analysis, performed in Patient-1 at 3 years of age, revealed a deeply altered glycosylation profile, with a three-fold increase of the monosialo- biantennary glycoform (A2G2S1) at *m/z* 2431.2 compared to the control (Fig. 4a) and the occurrence of an intense peak at 2227.1 (about 25% of the biantennary disialylated base-peak at 2792.4) corresponding to the aforementioned hypogalactosylated glyco-biomarker A2G1S1. Moreover, traces of truncated hyposialylated and/or hypogalactosylated glycoforms were detected at *m/z* 2070.1 (A2G2), at *m/z* 1865.9 (A2G1), at *m/z* 1661.9 (A2G0), together with traces of the hybrid glycan species at *m/z* 1982.0, the latter lacking the whole NeuAc-Gal-GlcNAc trisaccharide terminal branch. Unexpectedly, Patient-1’s transferrin glycosylation profile showed significant improvements over the years (Fig. 4c). In fact, a second serum transferrin analysis by MALDI-MS at 7 years of age, revealed a drastic reduction of the A2G1S1 N-glycan species, now present in trace amounts. Furthermore, the hypogalactosylated/hybrid species at *m/z* 1661.9, 1865.9 and 1982.0 were completely missing, supporting an age-related modification of serum transferrin glycosylation.

## Discussion

Here, we report the identification of novel *SCL39A8* variants and homozygosity for a common missense variant associated with clinical, neuroradiological and glycosylation findings consistent with SLC39A8-CDG. So far, a paucity of SLC39A8-CDG studies have been reported, including 14 patients with different clinical and neuroradiological characteristics and variable transferrin glycosylation changes [2–5] (Table 1). Common clinical features of SLC39A8-CDG patients are hypotonia, poor postural control, increased peripheral tone, global developmental delay, intellectual disability, and failure to thrive. Strabismus is a recurring feature [2–5], also in this study (Patient-2). Hearing impairment was described in only one patient [2]. Dysmorphic features (broad forehead, hirsutism, anteverted nostrils, thin lips, smooth philtrum) were reported in a few subjects [2, 4, 5, 10], including one infant with disproportionate dwarfism and craniosynostosis. Microcephaly was reported in one instance and cranial asymmetry was observed in Patient-2. Some patients affected by SLC39A8-CDG presented with scoliosis, as observed in the Patient-1, or with additional skeletal abnormalities [2–4]. In the present study, all patients were affected by dystonia and they also presented episodes of eye rolling and dyskinetic movements of the oral region. Due to feeding difficulties and poor weight gain, gastrostomy and fundoplication were performed in two study patients. Consistently, previous studies described patients with SLC39A8-CDG and prominent dystonic postural pattern who underwent gastrostomy [3–6]. According to what has been observed in the patients of this study and in others reported previously [5, 6], SLC39A8-CDG should be considered among CDG presenting with major hyperkinetic movement disorders such as global and segmental dystonia and dyskinesia, particularly at the orofacial region, accompanied by dysphagia [11]. Epilepsy is another common feature in SLC39-A8-CDG [2–5, 10] and it is often refractory to most anticonvulsant drugs [2, 4, 5]. Differently from Patient-1, who displayed atypical absences and clonic seizures, spasms with hypsarrhythmia [2, 5], tonic [5] and myoclonic seizures [3] have been reported.

**Table 1:** Clinical, molecular and glycosylation features in patients with SLC39A8-CDG

Patients ^[Ref.]^	1	2	3	4^1^	5^1^	6^2^	7^2^	8^2^	9^2^–10^2^		11^2^	12^2^–13^2^		14^3^–15^3^		16^4^	17^4^
Ethnicity	Italian		Romanian	German	German	Hutterite	Hutterite	Hutterite	Hutterite		Hutterite	Egyptian		Lebanese		Turkish	Hutterite
Sex/Age (years)	M/10	F/9	M/5	F/1	F/19	F/13	M/17	M/8	F/5	F/5	M/9	F/8	M/2	F/2	F/12	F/3	M/2
Progressive clinical course	+/+	+/+	−/−	−/−	−/−	−/−	−/−	−/−	−/−	−/−	−/−	−/−	−/−	+/+	+/−	+/+	+/−
Genotype	*c.1048G* > *A* *c.131C* > *G*		*c.1171G* > *A* *c.1171G* > *A*	*c.112G* > *C* *c.1019 T* > *A*	*c.97G* > *A; c.1004G* > *C/* *c.610G* > *T*	*c.112G* > *C* *c.112G* > *C*	*c.112G* > *C* *c.112G* > *C*	*c.112G* > *C* *c.112G* > *C*	*c.112G* > *C c.112G* > *C*		*c.112G* > *C/ c.112G* > *C*	*c.112G* > *C/* *c.112G* > *C*		*c.338G* > *C/* *c.338G* > *C*		*c.608 T* > *C* *c.608 T* > *C*	*c.112G* > *C* *c.112G* > *C*
Protein	G350A S44T		A391T A391T	G38R I340N	V33M; S335T G204C	G38R G38R	G38R G38R	G38R G38R	G38R G38R		G38R G38R	G38R G38R		C113S C113S		F203S F203S	G38R G38R
Serum transferrin IEF Type 2 pattern^°^	+	+/−	+/−	+	+	ND	+	ND	+		ND	ND		ND	+	Normal	Normal
Serum N-glycans Increased A2G1S1	+	+	+	+	+	ND	ND	ND	ND	ND	ND	ND		ND	ND	+	+
*Neurological features*																	
DD/ID	+/+	+/+	+/+	+/+	+/+	+/+	+/+	+/+	+/+	+/+	+/+	+/+	+/+	+/+	+/+	+/+	+/+
Microcephaly	−	−	−	−	−	−	−	−	−	−	−	−	−	ND	−	−	+
Hypotonia	+	+	+	+	+	+	+	+	+	+	+	+	+	+	+	+	−
Lower limb stifness	+	−	−	ND	−	−	−	−	−	−	−	−	−	+	+	−	+
Epilepsy (onset)	+(5 y)	−	−	+(4 m)	+(1 y)	−	−	−	−	−	+	+	−	+	+	+	+(5 m)
Dystonia/ Dyskinesia	+/+	+	+/+	ND	ND	ND	ND	ND	ND	ND	ND	ND	ND	+	+	+	+
Strabismus	−	−	+	+	+	+	+	+	+	+	−	+	+	−	+	+	−
Hearing loss	−	−	−	+	ND	ND	ND	ND	ND	ND	ND	ND	ND	ND	ND	+	−
Neuropathy	+	ND	−	ND	ND	ND	ND	ND	ND	ND	−	−	−	+	−	ND	ND
*Brain MRI changes*																	
Basal Ganglia	+	+	−	−	−	−	−	−	−	−	−	−	−	+	+	+	+
Thalamus	+	+	+	−	−	−	−	−	−	−	−	−	−	−	−	−	−
White Matter	+	+	+	−	−	−	−	−	−	−	−	−	−	−	−	+	−
Cerebral/ Cerebellar atrophy	+/+	+/+	−/−	+/−	−/+	−/+	−/+	−/+	−/+	ND	−/+	−/+	+/+	+/	+/−	−/−	−/−
*Systemic features*																	
Dysmorphisms	−	−	−	+	−	−	−	−	−	−	−	−	−	+	+	+	+
Skeletal changes	+	−	+	+	+	+	+	−	ND	ND	ND	ND	ND	−	+	ND	ND
Hepatopathy	+	−	−	+	ND	ND	ND	ND	ND	ND	ND	ND	ND	ND	−	+	−
Feeding difficulties	+	+	+	ND	ND	ND	ND	ND	ND	ND	ND	ND	ND	+	+	+	+

Others features: Atrial septal defect (Pt 16); Gastrostomy (Pts 1,2, 15,17); Skeletal abnormalities: Scoliosis (Pts 1,3,5,15), Craniosynostosis (Pt 4), Cranial asymmetry (Pt 3), Dwarfism (Pt 3), Osteopenia (Pt 6,7) Broadened long bone epiphysis (Pts 7); Joint hypermobility (Pts 5, 6, 7); Muscle Biopsy: Reduction of respiratory chain complexes I or I+III activity (Pts 1,2), Reduction of respiratory chain complex IV (Pts 14–15), Atrophic fibers with some subsarcolemmal lipid accumulation (Pt 14), Normal (Pt 2)

Age (years): age at last examination (years); F: female; M: male; m: months; ND: not determined; Pt(s): patient(s); y: years; +: present; −: Absent

Brain MRI patterns are highly variable in documented cases of SLC39A8-CDG (Table 1). Our patients presented two different distributions of lesions. Brain MRI in Patient-1 was characterized by a Leigh syndrome-like pattern with bilateral symmetrical basal ganglia hyperintensities and multiple focal signal alterations in subcortical white matter; a similar pattern was identified in the older sister as well (Patient-2) and in four other patients with SLC39A8-CDG irrespective of the underlying genetic variants [4, 5]. However, differently from previously described patients with deep grey matter lesions, Patient-1 and -2 also presented progressive cerebellar atrophy. Cerebellar atrophy is typical of several CDG and it was also found in seven of the eight cases reported by Boycott and colleagues [3] and in a patient described by Park and colleagues [2]. Interestingly, in Patient-1, MRS showed a lactate peak similar to that described in a patient with progressive atrophy of the cerebellar vermis and hemispheres [3]. Cerebellar involvement was not found in Patient-3, whose brain MRI documented thalamic volume loss, subcortical nonspecific alterations of signal, thinning of the corpus callosum and thickening of the anterior commissure but adequate progression of myelinisation and absence of cortical atrophy. Park and colleagues also described a CDG patient without cerebellar atrophy [2]; however, differently from Patient-3, in that case the neuroimaging was also characterized by brain asymmetry with cerebral atrophy of the left hemisphere and enlarged ventricles, especially on the left side of the brain [2]. So, judging from our cases and those already documented in literature, cerebral atrophy, cerebellar atrophy and a Leigh syndrome-like pattern are common features in SLC39A8-CDG and they can be present alone or in different combinations. However, no MRI finding can be considered pathognomonic of this CDG.

Muscular biopsies were performed in both Patient-1 and -3 for a suspected mitochondrial disorder, suggested by their clinical and radiological presentation. In Patient-1 a reduction of respiratory chain complex I activity was found, while respiratory chain enzymes in Patient-3’s skeletal muscle were suggestive of a low activity of complex I + III and an elevated activity of citrate synthetases. However, in both patients, the analysis of mitochondrial and nuclear genome did not show any variant related to mitochondrial disorders. Mitochondrial involvement in SLC39A8-CDG has not been systematically investigated by respiratory chain enzymology so far. However, low muscle complex IV and pyruvate dehydrogenase activity and low liver complexes IV and II + III were identified in a reported patient with Leigh syndrome-like presentation [4]. Thus SCL39A8-CDG should be sought in patients with features of Leigh syndrome-like mitochondrial disease that remain without a genetic diagnosis.

Extensive research on inducible global *Slc39a8*-knockout mice and *Slc39a8* liver-specific-knockout mice unequivocally showed that the hepatocyte ZIP8 transporter reclaims Mn from the bile, decreasing Mn biliary excretion and preserving Mn homeostatic levels in blood and tissues [1]. Disease-associated *SLC39A8* mutations caused retention of the ZIP8 transporter in the endoplasmic reticulum thus explaining the inability to localize at the plasma membrane and to transport Mn into cells [12]. Previous studies have investigated the reasons behind the reductions in respiratory chain complex activity in manganese-deficient conditions and have demonstrated that manganese deficiency leads to reduced activity of the reactive oxygen species (ROS) scavenger in mitochondria, MnSOD, manganese being its cofactor [13] and hence to increased levels of superoxide [14]. A direct study, however, demonstrated that *SLC39A8* disease-mutations reduced Mn levels in the mitochondria and, in turn, reduced mitochondrial MnSOD activity as well as mitochondrial function. Moreover, *SLC39A8* directly promotes the expression of several respiratory chain proteins and ATP production whereas *SLC39A8* mutations abolish these functions and enhance ROS generation [12]. ROS could damage mtDNA which encodes some subunits of complex I, III, IV and V of the respiratory chain, and could directly impair the activity of enzymes containing Fe-S clusters, as complex I, II and III [13].

Out of 14 patients with SLC39A8-CDG currently known, transferrin glycoforms analyses by IEF, CE or HPLC showed a CDG type 2 pattern in six [2–5] and normal patterns in two thus indicating that SLC39A8-CDG diagnosis may be missed by standard transferrin glycosylation analyses. In the patients of this study the sialotransferrin glycosylation patterns were characterized by variable and inconstant increases of trisialotransferrins and mild reductions of tetrasialotransferrins, consistent with the CDG type 2 pattern. We show for the first time in SLC39A8-CDG, age-related transferrin glycosylation changes documented by MALDI-MS in Patient-1. Having monitored transferrin glycosylation along the disease course we found an amelioration of hypoglycosylation in a 4-year period with spontaneous decrease of truncated glycans such as the hypogalactosylated form A2G1S1 at *m/z* 2227. Spontaneous amelioration of glycosylated biomarkers including transferrin has been reported in some other CDG such as PMM2-CDG [15] and SLC35A2-CDG [16]. Nevertheless, the findings of the present study should be taken into account when using transferrin hypoglycosylation changes to monitor the effectiveness of galactose and/or Mn supplementation therapy in patients with SLC39A8-CDG. Although transferrin glycosylation may only slightly abnormal (Patient-2 and Patient-3) or even normal in patients with SLC39A8-CDG [5], serum N-glycome analyses by MALDI-MS showed a distinct pattern in some previously studied patients [17, 18] (n:4) and in the patients here reported. In particular, total plasma N-glycome profiles are characterized by an increase of undergalactosylated and undersialylated precursors of fully sialylated biantennary glycans, especially the monosialo-monogalacto-biantennary glycan (A2G1S1, m/z 2227). In sum, comprehensive clinical, neuroradiological and glycosylation features are consistent with SLC39A8-CDG, supporting pathogenicity of the *SLC39A8* variants in our patients. In Family-1 we identified compound heterozygous Chr4(GRCh37): *c.1048G* > *A* (*p.Gly350Arg*) and *c.131C* > *G* (*p.Ser44Trp*) variants in *SLC39A8* that are not yet annotated in ClinVar. The *p.Gly350Arg* variant most likely modifies the splicing of on the 5’ splice site in exon 7. The *p.Ser44Trp* variant in exon 2 is located in the same position as the detrimental variant *p.Ser44Leu* according to GnomAD. Sanger sequencing confirmed the *SLC39A8* variants in both affected children and heterozygous in both parents. We here show, for the first time, that the clinical and glycophenotype of a homozygous *A391T* carrier (Patient-3) are consistent with SLC39A8-CDG. Taking into account the high allele frequency of the *A391T* (*p.Ala391Thr*) variant we can thus postulate a spectrum with a wide clinical variability among the variant carriers, ranging from undetectable asymptomatic/paucisymptomatic cases to more severe phenotypes likely due to a compound effect of modulator genes on a homozygous state.

The analysis of plasma protein N-glycosylation showed some degree of dysglycosylation in *A391T* (*p.Ala391Thr*) carriers, consistent with a significant increase of biantennary, and decrease of larger triantennary N-glycans and a trend towards less sialylated species than controls. An increase of A2G1S1 monosialo-monogalacto biantennary N-glycan (m/z 2227), consistently found in SLC39A8-CDG, was also observed in *A391T* homozygous carriers, although a complete clinical information on these subjects was not reported [1, 18]*.* The rs13107325 (C → T), was found to affect *SLC39A8* transcript levels in various tissues and may affect RNA levels in the brain [6, 18]. The *A391T* mutation is predicted to be disruptive to transporter function, though its precise location is unknown [19, 20].

To date there is little information about mutations related to SLC39A8-CDG (Table 1). Out of fourteen patients, *SLC39A8* variant (*c.112G* > *C, p.Gly38Arg*) homozygosity was found in nine individuals from three unrelated Hutterite families which establishes *p.G38R* as a pathogenic founder variant in the Hutterite population. Evolutionary alignment of *SCL39A8* amino-acid sequence showed a strict conservation of *Gly38* amino-acid in the protein. Several *SLC39A8* mutations such as *G38R* were found to affect the sequence motifs that control subcellular localization and were associated with retention of the ZIP8 transporter in the endoplasmic reticulum explaining the inability to localize at the plasma membrane and transport Mn into cells [12].

## Conclusions

The present study reports novel insights into the clinical, molecular and glycosylation features of SLC39A8-CDG. We document two novel variants associated with Leigh syndrome-like disease presentation of SLC39A8-CDG and we show, for the first time, a severe neurological phenotype in association with the already known homozygous *A391T* missense variant. We also found a spontaneous amelioration of transferrin N-glycome during the disease course and a distinct pattern of total serum N-glycome. This highlights the efficacy of MS-based serum glycomics for the diagnosis and clinical management of therapy response in patients with SLC39A8-CDG.

## Supplementary Information

**Additional file 1. Fig. S1**. Serum transferrin capillary zone electrophoresis in patients with SLC39A8 variants and healthy control. Patient -1 and -3 show an increase of 3-sialo transferrin,14,7% and 8% respectively (n.v.≤5.5%), and a decrease of 4-sialo transferrin, 75% and 82% respectively (n.v. ≥84%)

**Additional file 2. Fig. S2**. MALDI-TOF mass spectra of permethylated transferrin N-glycans in SLC39A8-CDG Patient-3 compared to a reference control. Transferrin N-glycan analysis in a pediatric representative control (**a**). Transferrin N-glycosylation analysis of Patient-3 (**b**), showing very slight unspecific glycosylation changes with minor increases of hyposialylated and fucosylated glycoforms. Compared to control, the observed changes are outlined by red marks. Glycan structures were assigned following consortium for functional glycomics guidelines: N-acetylglucosamine, blue square; mannose, green circle; galactose, yellow circle; sialic acid, purple lozenge; fucose, red triangle.

## Data Availability

Data are available upon requests to the corresponding author.
