# Supplementary figures and images for: Clinical, molecular and glycophenotype insights in SLC39A8-CDG
Source: Orphanet J Rare Dis. 2021 Jul 10;16:307. doi: 10.1186/s13023-021-01941-y (PMC8272319; doi:10.1186/s13023-021-01941-y)

## Slide 1
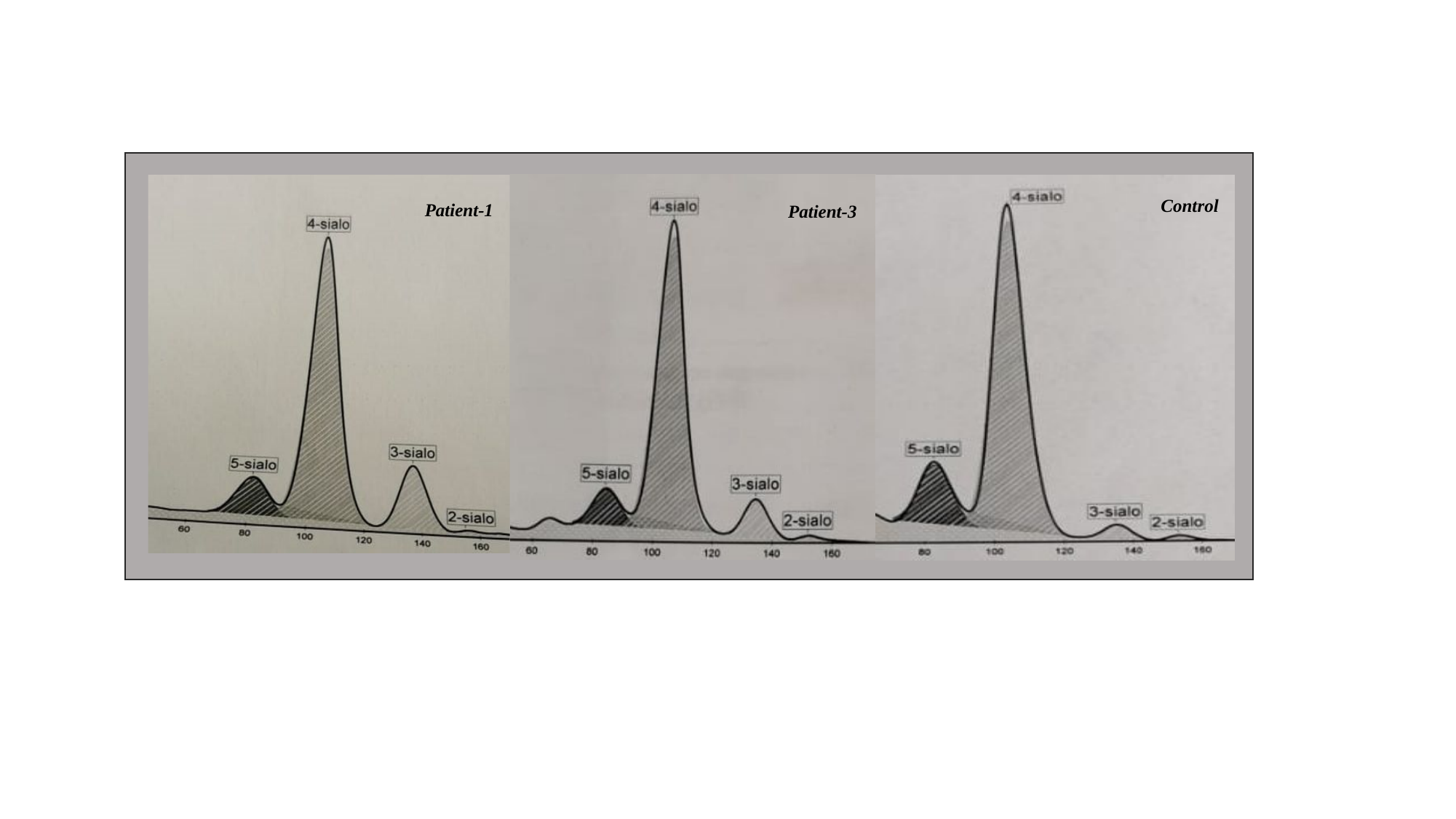

Control
Patient-1
Patient-3

Supplement: Supplementary file 1 — Additional file 1. Fig. S1. Serum transferrin capillary zone electrophoresis in patients with SLC39A8 variants and healthy control. Patient -1 and -3 show an increase of 3-sialo transferrin,14,7% and 8% respectively (n.v.≤5.5%), and a decrease of 4-sialo transferrin, 75% and 82% respectively (n.v. ≥84%) [file 13023_2021_1941_MOESM1_ESM.pptx]
