# Supplementary material for: Clinical, molecular and glycophenotype insights in SLC39A8-CDG
Source: Orphanet J Rare Dis. 2021 Jul 10;16:307. doi: 10.1186/s13023-021-01941-y (PMC8272319; doi:10.1186/s13023-021-01941-y)
Supplement: Supplementary file 2 — Additional file 2. Fig. S2. MALDI-TOF mass spectra of permethylated transferrin N-glycans in SLC39A8-CDG Patient-3 compared to a reference control. Transferrin N-glycan analysis in a pediatric representative control (a). Transferrin N-glycosylation analysis of Patient-3 (b), showing very slight unspecific glycosylation changes with minor increases of hyposialylated and fucosylated glycoforms. Compared to control, the observed changes are outlined by red marks. Glycan structures were assigned following consortium for functional glycomics guidelines: N-acetylglucosamine, blue square; mannose, green circle; galactose, yellow circle; sialic acid, purple lozenge; fucose, red triangle. [file 13023_2021_1941_MOESM2_ESM.pptx]

## Slide 1
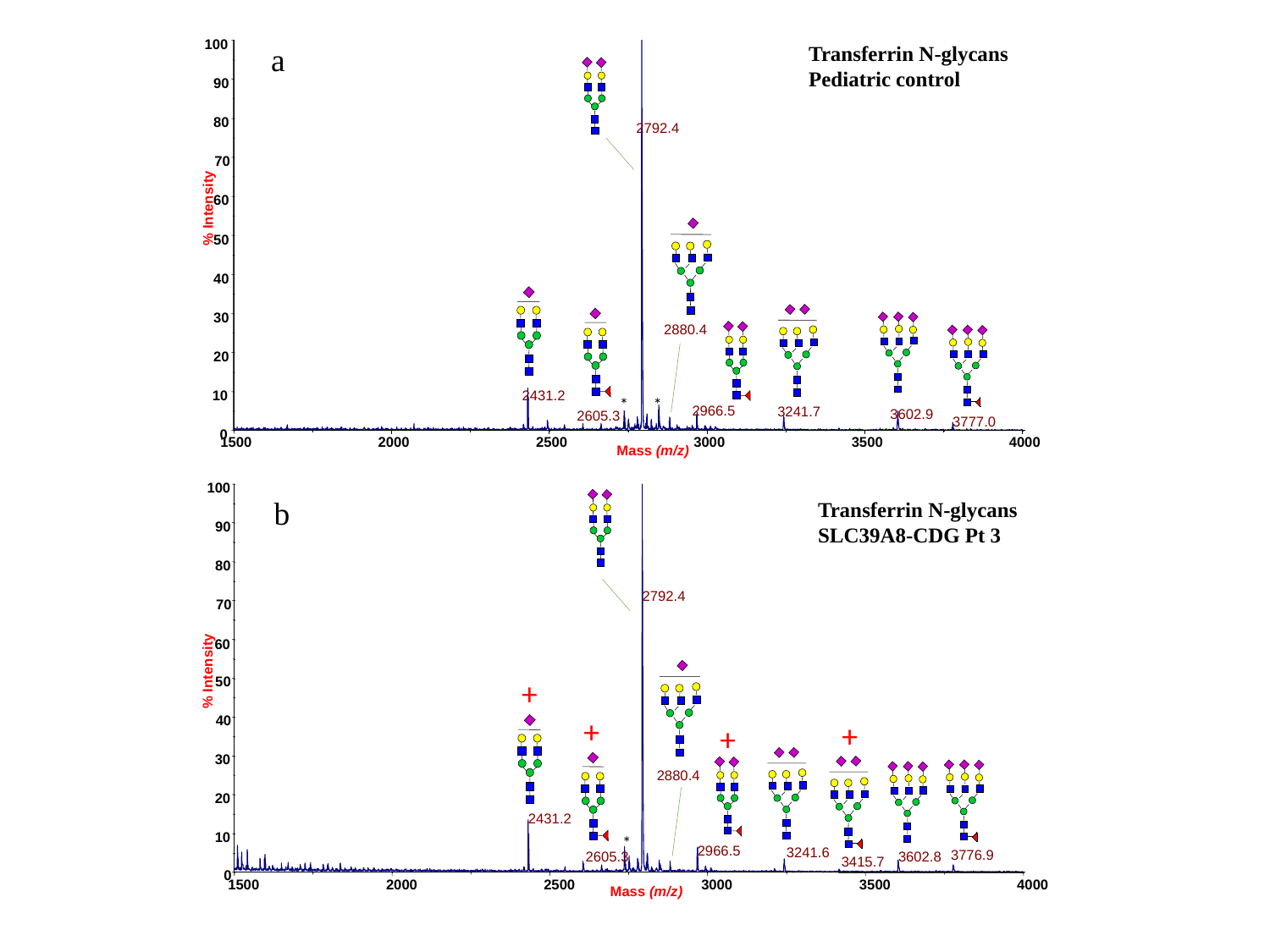

a
Transferrin N-glycans
Pediatric control
100
90
80
2792.4
70
60
% Intensity
50
40
30
2880.4
20
2431.2
10
*
*
2966.5
3241.7
3602.9
2605.3
3777.0
0
1500
2000
2500
3000
3500
4000
Mass (m/z)
100
b
Transferrin N-glycans
SLC39A8-CDG Pt 3
90
80
2792.4
70
60
% Intensity
+
50
+
+
40
+
30
2880.4
20
2431.2
*
10
2966.5
3241.6
3776.9
2605.3
3602.8
3415.7
0
1500
2000
2500
3000
3500
4000
Mass (m/z)
